# Patient-reported distress and correlates while on adjuvant endocrine therapy following breast cancer treatment among post-menopausal women: a group-based trajectory model analysis

**DOI:** 10.1007/s00520-026-10915-4

**Published:** 2026-06-25

**Authors:** Kara L. Gavin, Jacob Tiegs, Aaron N. Winn, Vaia Makris, Joan M. Neuner, Kathryn E. Flynn

**Affiliations:** 1 School of Medicine and Public Health, University of Wisconsin Madison, Madison, WI USA; 2 Center for Advancing Population Sciences, Medical College of Wisconsin, Milwaukee, WI USA; 3 Retzky College of Pharmacy, University of Illinois Chicago, Chicago, IL USA; 4 Department of Medicine, Medical College of Wisconsin, Milwaukee, WI USA

**Keywords:** Distress, Correlates, Breast cancer, Survivorship

## Abstract

**Purpose:**

To examine changes in patient-reported distress in female breast cancer (hormone-receptor-positive, stages 1–3) patients aged 55 + taking adjuvant endocrine therapy (AET).

**Methods:**

A retrospective cohort was constructed using the electronic health record (EHR) at a single Midwestern site from 2014 to 2019. Patients (*N* = 390) were included if they had completed two or more distress thermometer (DT) assessments. The DT is scored from 0 to 10; four is the threshold for clinically concerning distress. Trajectories were assigned using group-based trajectory modeling analyses. Predictors of group membership were identified using a cross-validated lasso algorithm.

**Results:**

The best-fit model included four groups. Group 1 (22%) had no distress, Group 2 (29%) had low distress (average DT = 0.8) that remained low, Group 3 (33%) had medium–low distress (2.7) that remained low, and Group 4 (16%) had high distress (6.2) that decreased but remained high (5) over time. Higher initial DT value was associated with a decreased odds of membership in Group 1 (OR = 0.88; 95% CI (0.80–0.95)) and increased in Group 4 (OR = 1.14; 95% CI (1.06–1.25)). Endorsement of sleep issues was associated with decreased odds of membership in Group 1 (OR = 0.59; 95% CI (0.40–0.87)) and increased odds of membership in Group 3 (OR = 1.53; 95% CI (1.11–2.26)).

**Conclusion:**

The majority of post-menopausal women do not report distress while taking AET; however, one in six reports high distress that did not dissipate with time. This work adds to the understanding of distress in breast cancer survivors and supports the use of the DT in providing actionable information on distress and its causes.

## Introduction

After completing primary treatments for hormone-receptor-positive breast cancer, patients, who are most often post-menopausal at diagnosis, are commonly prescribed 5–10 years of adjuvant endocrine therapy (AET) to prevent recurrence [1–5]. While the effectiveness of AET is well established, the side effects have led to high rates of non-adherence, with about 33% of patients discontinuing early [6]. Additionally, studies estimate that between one-quarter and one-half of those still taking pills are less than 80% adherent to the prescribed treatment regimen [1–5]. A predominant reason for non-adherence is the significant side effects that impact quality of life and increase distress [7]. The most common are menopausal symptoms, fatigue, and joint pain, with hot flashes reported most in patients on tamoxifen (55%) and arthralgias in patients on aromatase inhibitors (60%) [8–13].

It is common for breast cancer patients to experience distress, which may occur at any point from diagnosis to recovery [14]. Increased distress levels make it more difficult for women to cope with cancer diagnoses and treatments [14]. In a recent retrospective, longitudinal study, Lacourt et al. assessed distress levels monthly for 1 year, from the start of neoadjuvant chemotherapy through completion of chemotherapy, surgery, and radiation [15]. They found that distress was highest before starting treatment, and then decreased over time [16]. However, there is less understanding about changes in distress over time following active treatment, while patients are taking AET and potentially experiencing their associated side effects. A better understanding of the extent to which AET-related symptoms cause distress and how distress changes over time could help identify and prioritize targets for interventions to address patient distress and improve adherence to AET.

Distress is often measured using the NCCN distress thermometer (DT), a screening tool to measure distress in patients with cancer [14]. It has been used to identify correlates of distress in breast cancer patients and targets for intervention, including financial stress, anxiety and depression, and physical problems [17]. The DT is widely used in clinical settings throughout all phases of treatment, including by 84% of 55 large cancer centers [16], as it meets accreditation requirements for the Commission on Cancer. It is primarily given at an initial visit, and protocols for follow-up measurement vary, with many suggesting administration every 6 months [16]. When DT assessment is coupled with systematic interventions like mental health and social work referrals to address sources of distress, several outcomes improve, including communication and health services use during initial treatment [18, 19]. However, most studies using the DT during breast cancer treatment are cross-sectional and report only the prevalence of distress [20]. To identify different patterns of distress as women begin AET and continue through their course of treatment, this study aimed to examine trajectories of patient-reported distress in a retrospective cohort of older women over the first 2 years of AET treatment. We hypothesized that distinct groups would emerge based on DT scores (high vs. low). Exploratory analyses were then conducted to examine potential predictors of group membership.

## Methods

### Study design and cohort

We identified a retrospective cohort of breast cancer patients through the electronic health record (EHR) at a Midwestern academic cancer hospital between 2014 and 2019. We included patients based on the following criteria: female, aged 55 +, with stage I–III hormone-receptor-positive breast cancer, and reported to the North American Association of Central Cancer Registries (NAACCR). Women diagnosed prior to menopause often have more complicated treatments and more severe side effects of AET. We chose a cut-point of 55 years to ensure that women in the sample were post menopause. Additional eligibility criteria included at least one visit with a cancer center medical oncologist, a post-diagnosis prescription for AET (tamoxifen or the aromatase inhibitors anastrozole, letrozole, or exemestane) listed in the EHR, and at least one DT completed between 60 days before diagnosis and the first prescription for AET to establish a pre-AET distress level. We excluded patients with a diagnosis of dementia or those who used an investigational agent at the start of AET. The Medical College of Wisconsin Institutional Review Board reviewed and approved study procedures.

### Measures

We extracted all data from the EHR. The DT was administered to patients during routine clinical care every 6 months during oncology follow-up visits. The DT is a one-item measure scored 0–10, and the associated problem list includes 39 yes/no questions that comprise 5 domains: spiritual, family, physical, practical, and emotional. We dichotomized the score using the clinically accepted threshold where scores of 0–3 indicate low distress and scores of 4 or more indicate severe distress. To be included in the analysis, patients were required to complete at least two DT assessments: the first near the time of AET start and the second later in their treatment. We used the DT reported closest to the start of AET as the baseline for the pre-AET assessment. We used the maximum time from the AET DT assessment when multiple DT assessments occurred in the same 6-month interval. We also restricted DT assessments to instances where the patient had an ongoing record of AET medication; that is, we excluded DT assessments that occurred after a recorded discontinuation of AET medication. We identified AET discontinuation using the method described in our previous work [21].

#### Potential predictors of DT trajectory

Clinical variables included AET prescription information (Tamoxifen vs. AI), breast cancer treatment (radiation, chemotherapy, immunotherapy), HER2 status, Elixhauser comorbidity score, number of prescription medications, previous cancer history, mood disorder diagnoses including depression, anxiety or bipolar, and the PHQ-2 depression screener with a threshold of 3 [22]. Socio-demographic variables included age, race/ethnicity, insurance, and the area deprivation index of the zip code of residence [23].

### Analysis

#### Distress trajectories

We used group-based trajectory modeling (GBTM) to identify latent groupwise trends in the DT measures over a 2-year follow-up period [24, 25]. We conducted two separate analyses using observations from patients who provided 2 or more DT assessments. For each analysis, GBTM models were fitted with parameter adjustments to the degree of polynomial [1–3] and the number of groups [2–5] based on the DT score. We rejected models that yielded groups containing less than 10% (*n* = 39 observations) of the cohort. We selected a final best-fit model based on minimizing the Akaike information criterion (AIC) and Bayesian information criterion (BIC). We characterize the DT trajectory groups from the selected best-fit model. Additionally, we generated group membership predictions for each observation and used them in subsequent analyses to identify baseline predictors. 

#### Baseline predictors of trajectory membership

Treating the group trajectory assignment as the outcome, we identified baseline predictors for group assignment using a multinomial cross-validated (CV) lasso method [26, 27] nested within a 500-iteration bootstrap framework [27]. Within each bootstrap iteration, we completed the full CV lasso algorithm. We collected Lasso coefficient estimates from the one-standard-error lambda parameterization of the estimator. Additional parameterization included applying a grouped lasso penalization on the multinomial outcome, which produces symmetric coefficient estimates across the outcome groups. The symmetric multinomial odds ratio (OR) point estimates and 95% confidence intervals (CI) were calculated as the mean and 0.025 and 0.975 quantiles of the exponentiated bootstrapped coefficient distribution.

OR estimates were produced without using an established reference class from the multinomial outcome. Instead, a variable’s OR was produced for each multinomial class (i.e., a multinomial outcome with four classes would produce four ORs for a single variable). The inclusion proportion for each variable was also calculated as the proportion of bootstrap iterations in which the estimated coefficient was not equal to 0. An inclusion proportion threshold of 0.95 was used as an initial screening rule to identify potential predictors from the covariate set.

## Results

Female breast cancer patients (*N* = 390) had an average age of 68.4 years (SD = 7.7, range 55–90). During the 2-year follow-up, 266 patients completed two DT assessments, 116 patients completed three DT assessments, and 8 patients completed four DT assessments. The distribution of the completed DT indices was evenly spread across the 4 timepoints, with completion frequencies at the 6-, 12-, 18-, and 24-month time points of 234, 235, 225, and 218, respectively. Given the sharp drop in completion of three or more DTs, we completed analyses using two DT measures. We provide the overall cohort characteristics in Table 1.

**Table 1:** Cohort characteristics

	***N***
**Total**	390
**Time to AET therapy, in days, mean (SD)**	136.37 (73.24)
**Time to initial treatment, in days, mean (SD)**	34.75 (19.73)
**Time to systemic treatment, in days, mean (SD)**	94.28 (43.50)
**Age at date of diagnosis, in years, mean (SD)**	68.37 (7.67)
**Initial DT score, mean (SD)**	3.67 (2.91)
**Number of comorbidities, mean (SD)**	1.95 (1.69)
**Race, *n* (%)**	
Black	20 (5.1)
Other	6 (1.5)
White	364 (93.3)
**Ethnicity, *n* (%)**	
Non-Hispanic	387 (99.2)
Hispanic	3 (0.8)
**Insurance, *n* (%)**	
Medicaid	16 (4.1)
Medicare	238 (61.0)
Not insured	3 (0.8)
Other insurance	1 (0.3)
Private or commercial insurance	132 (33.8)
**HER2 status, *n* (%)**	
Equivocal	3 (0.8)
Negative	341 (88.8)
Positive	40 (10.4)
**PHQ 2 score < 3, *n* (%)**	372 (95%)

**Fig. 1:**
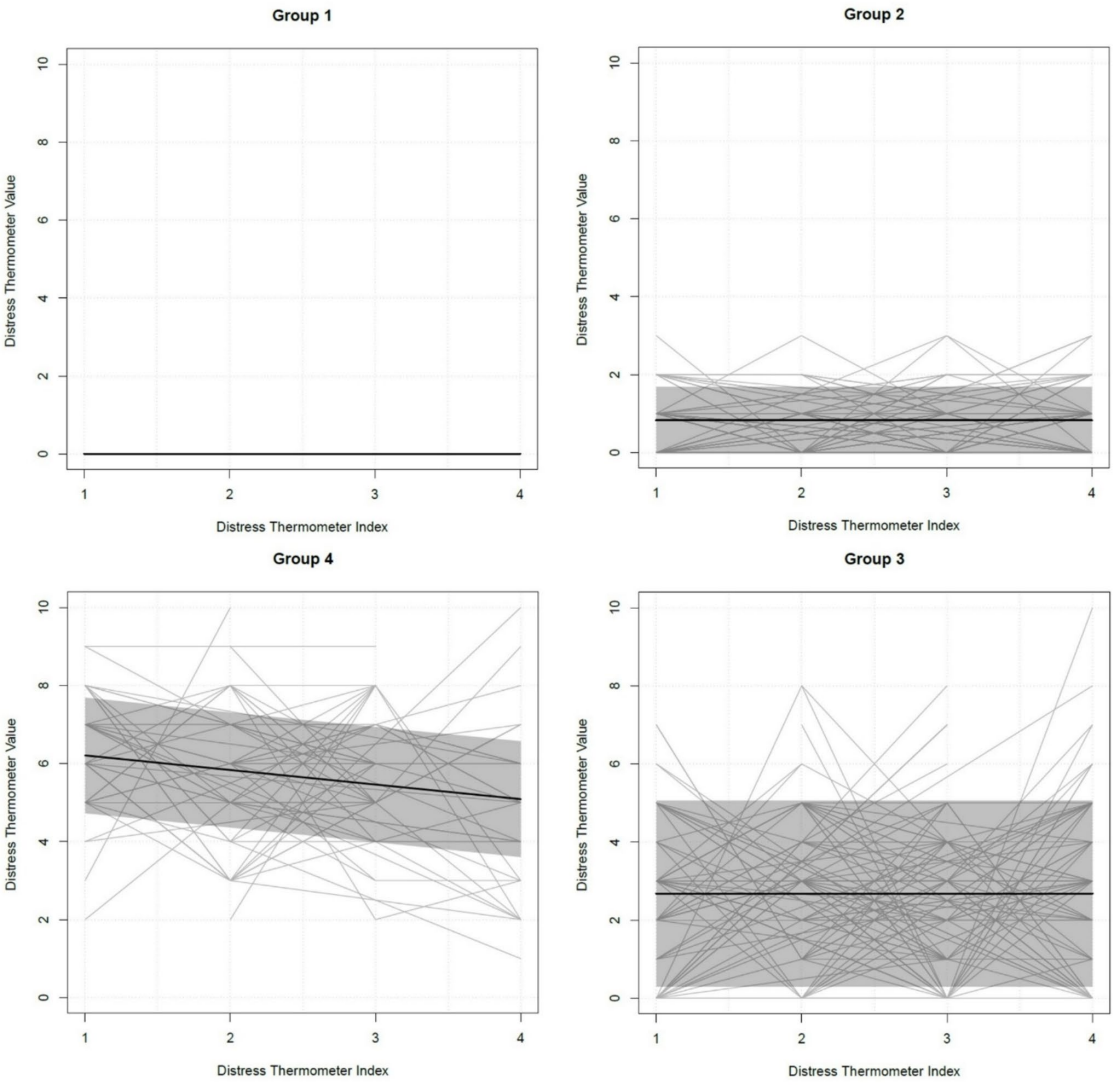
Changes in distress across the four different DT trajectory groups

The best-fit GBMT model included four trajectory groups (Fig. 1). Each group was well represented, with 22%, 29%, 33%, and 16% of the cohort assigned to Groups 1, 2, 3, and 4, respectively. Each group was well-defined and demonstrated distinct trends. Group 1 exhibited the simplest trend, characterized by no distress, with all DT assessments equal to 0; the standard error and prediction interval were also 0. Group 2 had low DT scores, with a mean DT value of 0.83 and a 95% prediction interval of 0–1.69, which did not change over the 2 years of follow-up. Group 3 had higher distress, but still below the clinically concerning threshold of 4, with a mean DT value of 2.68 and a wide 95% prediction interval of 0.30–5.06, which remained unchanged over the 2-year follow-up period. Group 4 exhibited high distress, with an initial (6 months) mean DT value of 6.21 and a 95% prediction interval of 4.72–7.69. This trajectory followed a decreasing trend of −0.37 units every 6 months, ending at 24 months with a mean DT value of 5.08 and a prediction interval of 3.60–6.58.

We identified baseline predictors from the bootstrapped lasso regression model (Table 2). From the possible 75 covariates, including 39 variables from the DT problem list, sociodemographic and clinical characteristics [21], 2 variables met the baseline 0.95 inclusion proportion criteria: baseline DT value and selecting issues with sleep from the DT problem list. A higher initial DT value was associated with a decreased OR for the DT trajectory Group 1 OR(CI) 0.88 (0.80–0.95) and an increased OR for DT trajectory Group 4, 1.14(1.06–1.25). Endorsement of an issue with sleep was associated with decreased OR for Group 1, 0.59 (0.40–0.87) and increased OR for Group 3, 1.53 (1.11–2.26) (Table 2). All other tested predictors did not reach statistical significance.

**Table 2:** Distress thermometer group trajectory associated predictors

Variable	Group	Odds ratio	95% Confidence interval	Inclusion proportion
Baseline diagnosis DT value	1	0.88	0.80–0.95	1.00
	2	0.98	0.94–1.03	0.98
	3	1.02	0.97–1.07	0.97
	4	1.14	1.06–1.25	1.00
Issues with sleep (true)	1	0.59	0.40–0.87	1.00
	2	0.98	0.74–1.30	0.99
	3	1.53	1.11–2.26	1.00
	4	1.14	0.87–1.53	0.99

Bootstrapped odds ratio (OR), 95% confidence intervals and inclusion proportion metrics for the three identified predictors. Metrics are included for each DT trajectory group. The OR is calculated without a base class and can be interpreted as odds of a single class versus odds of all other classes

## Discussion

Post-menopausal women on AET reported, on average, low levels of distress or distress that decreased over 2 years of follow-up. These findings are similar to and extend those in breast cancer patients undergoing neoadjuvant chemotherapy, who reported higher levels of distress prior to starting primary treatments with decreasing distress scores over time [15]. The GBTM analysis revealed that there are discernible trends in the DT assessments. Our analyses identified four groups as providing the best fit, with about half (51%) of the cohort falling into Groups 1 and 2, which were no or low distress with a 95% prediction interval below a DT value of 2. Another third (33%) of the cohort was assigned to Group 3. Group 3 was a medium–low trend line with a higher degree of variability, with a prediction interval of DT values of 0 to DT values of less than 6. The final 16% were in the high distress group. While this group showed a decrease in distress over time, the rate of decline corresponded to a 1 DT unit decrease on average over the 2-year follow-up period, and the average distress remained higher than the clinical threshold of 4 at all time points. Further, it showed that patients who had elevated distress (> 4) following the initiation of AET were likely to maintain high distress over the 2 years of follow-up. Similarly, patients with low distress (< 2) when starting AET were not expected to have high distress later. This suggests that administration of the DT could help to identify patients who are at risk for continued distress while on AET.

The subsequent analysis, which identified baseline predictors of distress trajectory groups, revealed two predictors associated with group membership. Higher initial DT values prior to beginning AET were associated with increased odds of having high distress (Group 4) and conversely with decreased odds of being assigned to the no-distress group (Group 1). These results are sensible given that those who experience low distress at baseline are more likely to continue to experience low distress over the next 2 years. Endorsement of *issues with sleep* was the only underlying symptom from the DT problem list identified as a predictor for DT group assignment, with a 41% decrease in odds of assignment to the no distress group (Group 1). However, the result did not indicate a strong association with increased odds of assignment to the high DT group (Group 4); instead, there was an increase in assignment to the mid–low DT group (Group 3).

This work adds to previous quality-of-life investigations in breast cancer patients receiving AET using patient-reported outcome measures, including the SF-12 and the PHQ-2 [28, 29], by identifying trends in patient distress after beginning AET. Of note, neither the SF-12 nor the PHQ-2, which measure patient mental health, was found to be predictors of distress trajectory. As both measures are used clinically to assess aspects of patient-reported health, our findings suggest that measuring distress offers important information to patient quality of life separate from overall health or depression and anxiety alone. Further, that only two of the 54 potential covariates met the threshold to be included as a predictor of distress trajectory assignment highlights the importance of examining change over time. Previous work that has examined correlates of distress has typically measured both distress and its correlates at one time point. This work broadens our understanding of distress in older breast cancer survivors by examining how distress can change over time and what might impact that.

Our work emphasizes the utility of the DT measure, which can provide actionable information on distress severity and specific problems, and suggests that DT scores before starting AET may identify patients at the highest risk for sustained distress. While these trajectories themselves do not highlight specifics about what can be done to help patients who have high distress, interventions can be created around asking people about specific causes of distress and offering management strategies for those. Recent findings of Rosso and others found that 84% of patients discontinued AET due to side effects and another 11% discontinued for an unspecified intolerance [11]. While this study does not directly address adherence, our findings suggest that about one in six patients has continued distress over time. As AET is now recommended for longer periods, up to 10 years for many women, monitoring distress may be one strategy for identifying patients for further assessment and intervention. For example, patients who continually report musculoskeletal pain or fatigue may benefit from guideline-concordant supportive treatments offered earlier on. Future studies should examine whether interventions that seek to improve physical symptoms and distress across domains, like the ones reported in the DT, are successful in improving long-term adherence and continuation of AET out to 10 years when recommended.

These findings should be considered in light of several limitations, including limited racial and ethnic diversity in the cohort. The retrospective data for this study were collected at one academic medical center in a sample of older women, the majority of whom were not depressed based on the PHQ-2 screener, which may further limit generalizability. Additionally, while patients could have completed up to four DT assessments, the majority of the sample only completed two. This limited our ability to detect more complex patterns of change over time and to pinpoint, for those who experienced changes in distress, when distress levels changed; thus, later changes in distress may not have been detected. Furthermore, it is possible that patients with high distress or very low distress may not have completed the DT measure imposing a bias on our identified trends. This highlights the need for regular measurement and reporting of DT scores in the electronic health record to monitor for increases in patient distress. This may be challenging as patients are seen less frequently in the clinic than during active treatment. Still, remote monitoring may be a promising option, since the DT is relatively short and easy for most patients to complete and the problem list helps to identify specific sources of distress that may be treatable through supportive care. Additional work and regular monitoring are needed to better understand sources of distress and identify which patients should be offered interventions.

## Data Availability

The data that support the findings of this study are available from Medical College of Wisconsin, but restrictions apply to the availability of these data, and so are not publicly available.
